# Human helminth infections above latitude 60°N: reports published 2001−2024

**DOI:** 10.1017/S0031182025100577

**Published:** 2025-08

**Authors:** Tapan Bhattacharyya, Michael A. Miles

**Affiliations:** Faculty of Infectious & Tropical Diseases, London School of Hygiene and Tropical Medicine, London, UK

**Keywords:** Alaska, Anisakid, Canada, *Dibothriocephalus*, *Echinococcus*, Greenland, *Opisthorchis*, Russian Federation, *Toxocara*, *Trichinella*

## Abstract

This article surveys reports of human helminth infection from geographical regions above latitude 60°N published in the period 2001–2024. We take a global approach encompassing the Americas and Eurasia. The helminth genera thus described herein include nematode (*Trichinella, Toxocara, Anisakis, Pseudoterranova*), cestode (*Echinococcus, Dibothriocephalus*) and trematode (*Opisthorchis, Trichobilharzia*). The primary reports identified infections principally by serology (community-based or individual, including imported cases) and outbreaks. There were also articles reporting national data compiled from official sources. Despite successful local control programmes, these pathogens pose an ongoing risk to human health in this region.

## Graphical abstract

**Figure:**
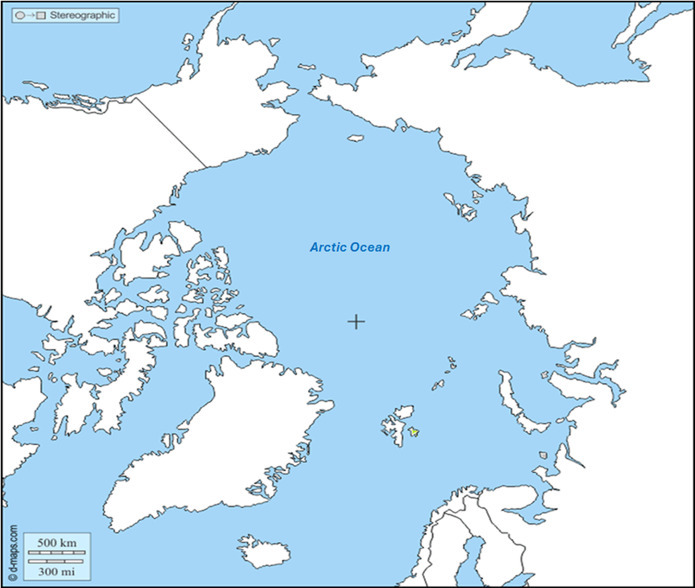

## Introduction

This article surveys reports of human infection with parasitic helminths in the global Arctic and sub-Arctic region, northwards from a latitude of approximately 60°N, published in the period 2001–2024. We use 60°N to focus on the northernmost part of the inhabited world as it has relatively sparse literature compared to other regions of the world.

Previous review articles in the literature have concentrated on particular helminth genus or geographical region, for example: *Trichinellosis* (Ozeretskovskaya et al., 2005; Oksanen et al., 2022); *Echinococcus* (Davidson et al., 2016); *Dibothriocephalus* (Kralova-Hromadova et al., 2021; Kuchta et al., 2023); the Americas (Jenkins et al., 2013). Those articles have also incorporated historical perspectives. Generally, there has been a decline in human helminth infections in this region during the 20th century, due to public health campaigns and specific control and monitoring programmes. These include for *E. granulosus* in Iceland (Saarma et al., 2023) and Finland (Hirvelä-Koski et al., 2003; Oksanen and Lavikainen, 2015) and *E. multilocularis* surveillance in Nordic countries (Wahlström et al., 2015).

Here, by applying a 21st-century timeframe and a global lens, we concentrate on more recent reports to highlight the ongoing risk of human infection.

## Methods

Search terms comprising genus name and geographical region, e.g. [‘*Trichinella*’ AND ‘Human’ AND ‘Greenland’] were searched on PubMed, Web of Science and Google Scholar for publications dated from 2001 onwards. There were no language restrictions. Excluded were articles in which the source of infection was identified or suspected by those publications’ authors as below 60°N.

## Results

Of the articles identified for inclusion herein, the majority (*n* = 27) were primary reports of helminth infection that were either fully accessible to us online or in a few cases only the Abstract. There were a smaller number of publications that compiled national sources of data and/or unpublished reports for which the primary data were not readily attainable.

The helminth genera comprised nematodes *Trichinella, Toxocara, Anisakis, Pseudoterranova*, cestodes *Echinococcus, Dibothriocephalus* and trematode *Opisthorchis, Trichobilharzia*. All are zoonotic and, with the exception of *Trichobilharzia*, are acquired by ingestion of infective helminth propagules from undercooked game (mammal or fish) or by exposure to materials contaminated by animal faeces (Benesh et al., 2021). The identified articles concentrated on a single genus or considered up to 3 genera.

**Figure 1.**
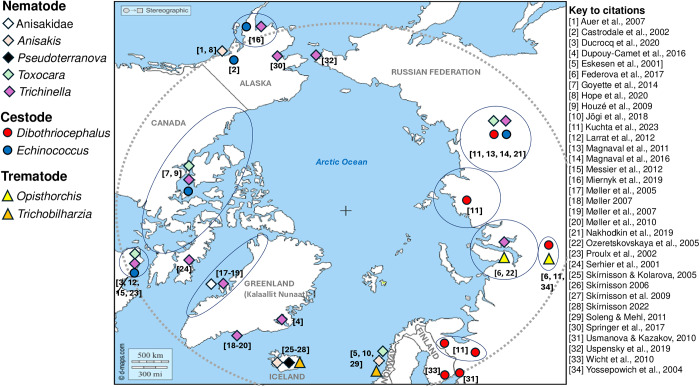
Indicative locations of the studies surveyed herein. Encircled regions show the geographical scope of regional studies. Dashed line represents latitude 60°N.

Geographic locations in the Americas were in Alaska, Canada and Greenland (Kalaallit Nunaat), and in Eurasia were in Iceland, Norway, Finland and Russian Federation (Figure 1). In the primary reports, infections were identified by serology in community surveys (Table 1) or in individual cases (sometimes with parasitological or molecular demonstration), or as part of outbreaks (principally of *Trichinella*, Table 2).

**Table 1.** Geographical distribution of seroprevalence studies surveyed herein

			Reported seroprevalence (%)			
Region	Site(s)	Year of sampling	*Trichinella*	*Echinoccoccus*	*Toxocara*	Reference
Alaska	Various, southern Alaska	2007−2008	5	1.9	–	(Miernyk et al., 2019)
Canada	Nunavik, northern Quebec	2004	≤1	8.3	3.9	(Messier et al., 2012)
	Coastal Nunavut	2007 & 2008	18.6	6.3	1.7	(Goyette et al., 2014)
Greenland	Mainly western Greenland	1979−1981 & 1998−2004	^a^	–	–	(Møller, 2007)
	Western Greenland	2001	1.1	–	–	(Møller et al., 2007)
	Ammassalik municipality, East Greenland	2004	3.1	–	–	(Møller et al., 2010)
Norway	Bergen area	2010–2015	–	–	11.7	(Jõgi et al., 2018)
Russian Federation	Sakha/Yakutia, north-eastern Siberia	2007	4.4	0	4.4	(Magnaval et al., 2011)
	Sakha/Yakutia, north-eastern Siberia	2007−2008	–	–	3	(Akhmadishina et al., 2020)
	Bering Sea settlements, Chukotka	2010 & 2011	24.3	–	–	(Uspensky et al., 2019)
	Sakha/Yakutia, north-eastern Siberia	2012	0	1.3	0	(Magnaval et al., 2016)
	Sakha/Yakutia, north-eastern Siberia	2018	2.2	4.4	1.1	(Nakhodkin et al., 2019)

a See text.

**Table 2.** Trichinellosis outbreak clusters surveyed herein

Region	Site	Year	Cases	Authors’ remarks	Reference
Alaska	Norton Sound area	2016 & 2017	10	Walrus meat consumption implicated in 2 outbreaks	(Springer et al., 2017)
Canada	Nunavik, northern Quebec	1997	3	A control programme prevented a larger outbreak caused by walrus meat consumption	(Proulx et al., 2002)
	Coastal Nunavut	1999	7	Walrus meat consumption	(Serhir et al., 2001)
	Kuujjuaq, Nunavik	2004	3	Black bear meat consumption	Cited in (Larrat et al., 2012)
	Kangiqsujuaq, Nunavik	2006	2	Walrus meat consumption outside Nunavik	Cited in (Larrat et al., 2012)
	Victoria Island, Nunavut	2009	5	Grizzly bear consumption by travellers most likely source	(Houzé et al., 2009)
	Nunavik, northern Quebec	2013	18	Precise cause not identified, distributed polar bear meat considered most likely.	(Ducrocq et al., 2020)
Greenland	Western Greenland	2001	6	Walrus meat most likely source	(Møller et al., 2005)
	Eastern Greenland	2016	3	Polar bear meat consumption by travellers	(Dupouy-Camet et al., 2016)
Russian Federation	Yamal-Nenets, north-western Siberia	2002	10	Brown bear meat consumption	Cited in (Ozeretskovskaya et al., 2005)

The highest number of articles were on *Trichinella*, followed by *Toxocara* and *Echinococcus*. Articles on the trematodes *Opisthorchis* and *Trichobilharzia* referred only to Eurasia, whereas the other genera were represented by articles referring to both Eurasia and the Americas.

In the following sections, each genus is considered in turn, with a brief description of propagule infection followed by the reports according to geographical setting.

### Nematodes

#### Trichinella *spp.*

Consumption of inadequately cooked game meat containing parasite larvae can lead to diarrhoea, fever, myalgia, facial oedema, eosinophilia and elevated levels of muscle enzymes. The predominant regional taxon of this genus is encapsulated *T. nativa*, viable at low (frozen) temperatures (Pozio, 2016).

**Americas**. A serosurvey among bird-hunting populations of southern Alaska sampled in 2007–2008 found 5% seropositivity, although no specific source of infection was proposed (Miernyk et al., 2019). However, outbreaks of trichinellosis related to the consumption of walrus (*Odobenus rosmarus*) were identified in 2016 and 2017 in the Norton Sound area of western Alaska. In the 2016 outbreak, 5 members of the same family were affected; the following year an outbreak was suspected in a separate community, involving 5 neighbours who had shared walrus meat. In both outbreaks, the incriminated meat was not available for testing, but stored (frozen) meat from the household of the 2017 outbreak was identified by polymerase chain reaction (PCR) to contain *T. nativa* (Springer et al., 2017).

A study in 2004, part of a Canadian health survey in the northern Quebec region of Nunavik, reported a seroprevalence of ≤1% for the 14 sites as a whole (Messier et al., 2012). In contrast, a study conducted in 2007 and 2008 across coastal regions further north in Nunavut, as part of the International Polar Year Inuit Health Survey, reported a combined seroprevalence of 18.6% from >30 communities; these remote far northern study populations were reached by sea or air (Goyette et al., 2014). An analysis of Canadian hospital admissions between 2001 and 2005 revealed that there was a much higher incidence in Nunavut and Nunavik together than in the rest of Canada combined (Gilbert et al., 2010).

Outbreak clusters in northern Canada reported since 2001 have been associated with coastal communities. In 1997, the implementation of a novel control programme including PCR identification of *T. nativa* in walrus meat samples and community engagement prevented a larger outbreak in Puvirnituq and Inukjuak (west coast of Nunavik) (Proulx et al., 2002). In 1999, 7 individuals, who had eaten raw walrus meat a few weeks previously, presented at a health centre in Qikiqtarjuag (eastern coast of Nunavut) with symptoms including diarrhoea, abdominal pain, rash and swelling. All were found to have high levels of anti-*Trichinella* antibodies and eosinophilia (Serhir et al., 2001). In 2009, a cluster in a group of sailors returning to France after exploring the Northwest passage on 2 different boats was considered to be most likely caused by consumption of frozen meat of grizzly bear (*Ursus arctos*) around Cambridge Bay (Iqaluktuuttiaq), Victoria Island, Nunavut. Prior to definite diagnosis, 2 of the sailors while still on-board experienced influenza-like symptoms (Houzé et al., 2009). In 2013, another outbreak in Inukjuak involved 18 individuals across 15 different households, notable as most of the cases were adult women and also as the investigators were not able to identify a specific event or food source as a cause, although distributed meat of polar bear (*Ursus maritimus*) was considered the most likely (Ducrocq et al., 2020).

A seroprevalence study comparing samples from various settlements predominantly in western Greenland archived between 1979–1981 and 1998–2004 identified (i) game meat consumption as the main risk factor for infection and (ii) a declining trend in seropositivity between the 2 time periods, likely due to the increased consumption of industrially produced food (Møller, 2007). A seroprevalence of 1.1% was found from a 2001 survey among children under 14 years of age in western Greenlandic settlements (Møller et al., 2007). Similarly, a 2004 sampling among game hunting communities in eastern Greenland revealed an increase in seropositivity according to age, 1.4% vs 7.5% in participants aged under or over 40 respectively; seroprevalence for the ⩾60 years age group alone was 12%. ‘Occupation hunter/fisherman’ and ‘polar bear meat consumption’ were identified as significant risk factors (Møller et al., 2010).

As elsewhere in the Americas, outbreak clusters have been recognized in Greenland in recent decades. In 2001, a cluster associated with consumption of game meat, suspected to be walrus, was identified near Aasiaat in west Greenland. Serological confirmation by ELISA and Western blot was achieved in 4 of the 6 cases initially; 1 of the 2 seronegative cases sero-converted when tested a year later, the authors considered this due to new infection (Møller et al., 2005). In a later outbreak, 3 travellers returning to France in 2016 presented at a Paris hospital with symptoms including myalgia, diarrhoea, with elevated eosinophilia and creatinine kinase levels. They had consumed meat of polar bear in eastern Greenland a few weeks before; trichinellosis was confirmed by ELISA and Western blotting (Dupouy-Camet et al., 2016).

**Eurasia**. In the Russian Federation, sampling in 2007 from Viljujsk city, Sakha/Yakutia, north-eastern Siberia, reported 4.4% seroprevalence by ELISA (Magnaval et al., 2011). From sampling in 2 coastal settlements in Chukotka on the Arctic coast of the Bering Sea in 2010 and 2011, 24.3% seroprevalence was found by ELISA using in-house-generated *T. nativa* excretory–secretory antigen, with important sources of infection being meat of walrus and seal (*Phoca*) (Uspensky et al., 2019). A serosurvey in 2018 in rural areas of Central Sakha/Yakutia used commercial ELISA kit to detect 2.2% seroprevalence; the authors also assessed IgG against *E. granulosus* and *T. canis*, as described in the sections below. However, in that study, none of the 3 helminths had correlations with possible exposure variables identified (Nakhodkin et al., 2019).

A listing of outbreaks within the Russian Federation between 1996 and 2002, compiled from official sources, cites an outbreak involving 10 cases in Yamal-Nenets in north-western Siberia caused by consumption of the meat of the brown bear (*Ursus arctos*) (Ozeretskovskaya et al., 2005).

#### Toxocara *spp.*

Ingestion of embryonated eggs in material contaminated with dog or cat faeces results in the release of larvae that migrate in the human body, consequently, toxocariasis comprising several pictures (covert toxocariasis, visceral larva migrans, ocular or neurological toxocariasis) may occur.

**Americas**. The Canadian studies of Messier et al. and Goyette et al. described above reported 3.9% and 1.7% seroprevalence respectively for human toxocariasis using a commercial ELISA (Messier et al., 2012; Goyette et al., 2014). Decrease in seropositivity associates with northerly latitude in Canada (Bradbury and Panicker, 2020).

**Eurasia**. Anti-*Toxocara* IgG4 seroprevalences of 17.5% and 8.0% in parents and offspring respectively were found as part of a Norwegian inter-generational study (overall 11.7%), identifying positive associations with allergic symptoms among the offspring. The authors described a soluble worm somatic antigen preparation for use in the ELISA (Jõgi et al., 2018).

Seroprevalence has been reported from Sakha/Yakutia (Russian Federation). In a 2007 sampling, seroprevalence by Western blot of 4.4% was reported in a Northwestern area (Magnaval et al., 2011), although none was found in other settlements of the Far North (Magnaval et al., 2016). In line with this, a sampling in in 2007–2008 revealed 3% seroprevalence by commercial ELISA, among the lowest in the geographical range (south to north) of Russian regions examined (Akhmadishina et al., 2020), and a 2018 sampling identified 1.1% seroprevalence (Nakhodkin et al., 2019).

#### Anisakids

Larvae of anisakid species such as *Anisakis simplex* and *Pseudoterranova decipiens* in muscle of undercooked marine fish are released by human stomach enzymes, leading to gastric or intestinal infection. Both locally acquired (Norway, Iceland, Greenland) and imported cases (from Alaska) have been reported.

**Americas.** The study of western Greenlandic settlements mentioned above for *Trichinella* also reported the first human arctic IgG against Anisakidae (Møller et al., 2007). In imported cases, anisakiasis was detected histologically within an inguinal hernia in a patient who had consumed raw salmon from a stream on a recent fishing trip to Alaska (Hope et al., 2020). The first documented imported cases in Austria occurred in 2 travellers returning from an Alaskan fishing trip where they had consumed cold smoked salmon. Diagnosis was by specific antibody detection, the morphological and molecular identification of *A. simplex s. str*. larvae in the consumed salmon and of *Anisakis* DNA in the resected ileum of 1 of the patients (Auer et al., 2007).

**Eurasia**. In Norway, a chronic infection was identified by decreased *A. simplex* IgE serology following resection of an occluding duodenal tumour, and suggestive identification of a tubular sclerotic structure 1–2 mm in diameter. The authors reported the patient’s history of consumption of prepared saltwater fish (Eskesen et al., 2001).

Between 2004 and 2020 in Iceland, 16 human cases of *P. decipiens* and 2 of *A. simplex* were identified in this island nation (Skírnisson, 2022). Among these, *P. decipiens* larvae were found in the throats of patients a few days after consumption of inadequately cooked catfish (Skírnisson, 2006).

### Cestodes

#### Echinococcus *spp.*

Transmission is by ingestion of eggs in material contaminated with canid faeces (leading to hydatid cyst), or following contact with foxes (alveolar echinococcosis, AE).

**Americas**. Two documented human cases of *E. granulosus* cystic echinococcosis (CE) in Alaska were reported as having unusually severe presentations (Castrodale et al., 2002). The 2007–2008 sampling mentioned above (Miernyk et al., 2019) reported 1.8% and 0.1% seroprevalences for *E. granulosus* and *E. multilocularis*, respectively.

The Canadian studies of Messier et al. and Goyette et al. described above reported 8.3% and 6.3% seroprevalence respectively for *E. granulosus* only (Messier et al., 2012; Goyette et al., 2014). Analyses of Canadian hospital admissions have also been undertaken. One study of the years 2001–2005 revealed the incidence for echinococcosis increased with northerly latitude, the highest being from above 55°N. The type of echinococcosis (CE or AE) was not specified (Gilbert et al., 2010). A different analysis for year range 2000–2020 also found that Northwest Territories, Nunavut and Yukon together (all above 60°N) had a much higher risk of echinococcosis (relative risk 17.1; 95% confidence interval: 8.7–33.7) compared to more southerly Atlantic provinces, with Northwest Territories having the highest national risk and increase (6.3–9.1 cases/million) but decreases in Nunavut and Yukon (8.6–2.6 and 5.3–5.1 respectively/million). The number of identified cases of *E. granulosus* and *E. multilocularis* for Canada as a whole were given by those authors, but not specified geographically for each province (Khalid et al., 2024).

**Eurasia**. Sampling in 2012 in the Russian Federation identified 1.3% seroprevalence of AE in a study of 2 villages in the Verkhoyansk district (Far North Sakha/Yakutia) by ELISA using commercial soluble extract of *E. granulosus* protoscoleces and a second-tier Western blot using *E. multilocularis* whole larval extract for AE discrimination (Magnaval et al., 2016). A 2018 sampling in the same region reported 4.4% seroprevalence (Nakhodkin et al., 2019).

#### Dibothriocephalus *spp.*

Following ingestion of the plerocercoid from undercooked fish, adult worms may grow to several metres in length within the human host.

**Americas**. A report of traveller returning to Austria, who passed a 75 cm tapeworm segment in stool, was suspected to have been infected during an Alaskan fishing tour 14 months earlier. The otherwise asymptomatic patient, without weight loss, anaemia or eosinophilia, was treated successfully with a single dose of praziquantel (Stadlbauer et al., 2005).

**Eurasia**. A report of molecular typing of *D. latus* DNA included an infection from a Finnish patient, who reported having eaten a local fish meal in the southern coastal locality of Kotka (Wicht et al., 2010).

An analysis of official reports and Russian-language sources revealed information according to region. North-western Russia: in Karelia nearly 300 cases between 2011 and 2013; in Arkhangelsk a decrease in incidence/100 000 from 6.99 to 2.74 between 2006 and 2017. Ural district: in Khanty-Mansi region 210 cases between 2020 and 2022. Siberia: incidence/100 000 in Evenk (548.8) and Taymyr Dolgano-Nenets (343.7) administrative regions were the highest nationally. Far East: in Sakha/Yakutia decrease in incidence to 112.2/100 000 by 2016 (Kuchta et al., 2023). Molecular analysis of an adult worm expelled from a patient in the St Petersburg area allowed the identification of repetitive elements in the *D. latus* genome (Usmanova and Kazakov, 2010).

### Trematode

#### Opisthorchis *spp.*

Following ingestion of metacercaria from undercooked fish, adult worms live in human bile ducts, significantly increasing risk of cholangiocarcinoma in chronic infection.

**Eurasia**. Infection with *O. felineus* is endemic in many parts of Russian Federation, principally in Western Siberia. An analysis of official sources for 2011–2013 revealed the highest incidence in Khanty-Mansi and Yamal-Nenets regions (599.7 and 261.9 cases respectively/100 000/year). Approximately 30 000 new cases were diagnosed each year nationally (Fedorova et al., 2017).

A familial outbreak in Israel was traced to imported fish eaten 10 days earlier that had been originally bought in Nizhnevartovsk, Khanty-Mansi region. Diagnosis was confirmed by identification of worm ova in patient stool, and presumed to be *O. felineus* due to high levels of endemicity in the region of origin (Yossepowitch et al., 2004).

#### Trichobilharzia *spp.*

These members of the Schistosomatidae family infect humans through skin penetration by the cercariae which emerge from the intermediate snail host. Cercarial dermatitis (‘swimmers itch’) is caused by zoonotic schistosomes which do not develop fully in humans. Following infection, a pruritic dermal maculopapular response develops.

**Eurasia**. In Iceland, outbreaks of cercarial dermatitis after infection by bird schistosome cercariae have been reported since 2000 (Skírnisson et al., 2009). Two outbreaks occurred in a touristic geothermally heated brook in Landmannalaugar in the southern interior of the island in 2003 and 2004. The authors state that these were caused by increased numbers of *Trichobilharzia* schistosomes deriving from mallard (*Anas platyrhynchos*) ducklings and developing in *Radix peregra* snails (Skírnisson and Kolarova, 2005).

In a compilation of reports from Norway between 2001 and 2009, cercarial dermatitis was recorded in dozens of lakes throughout the length of that country (Soleng and Mehl, 2011). The authors of that study identified *Trichobilharzia franki* cercariae shed from a *R. auricularia* snail in a sampling in 2008.

## Discussion

The prevalence of neglected infectious diseases in Arctic and sub-Arctic communities may not always have been well considered in global disease burdens, due to factors such as the geographical and societal remoteness of such populations (Hotez, 2010).

Our intention here was to survey recent literature for a broad range of human helminths (nematode, cestode and trematode) to encompass the inhabited global region above 60°N. From fully accessible (online) primary reports, we could extract details including locations, sampling, and diagnostic tests. However, this was not always possible, as some articles referred to compiled national data (Ozeretskovskaya et al., 2005; Gilbert et al., 2010; Fedorova et al., 2017; Khalid et al., 2024; Skírnisson, 2022; Kuchta et al., 2023) or only the abstract was accessible to us (e.g. Auer et al., 2007; Skírnisson, 2006).

Many of these studies used commercial ELISA tests to identify immunoglobulin G (IgG). Some authors acknowledged the possible limitations of the commercial ELISAs, such as used for *Trichinella*, including lack of specificity, and confounding longevity of IgG, as caveats (Messier et al., 2012; Goyette et al., 2014). For *Toxocara*, ELISA or Western blot using *T. canis* excretory-secretory antigens do not discriminate between infection due to *T. canis* and that due to *T. cati*. Such considerations may also skew associations with risk factors. Therefore, the identification and characterization of outbreak clusters becomes of particular interest. Those described here principally relate to trichinellosis, for which the most literature was readily accessible, predominantly from the Americas. These demonstrate that transmission to humans is ongoing and therefore almost certainly under-reported due the isolation of hunting communities or mild or unrecognized symptoms.

Among reports in returning travellers, infections originated in the Americas (Alaska, Canada, Greenland). These articles reinforce the importance of travel history including food consumption, which may otherwise lead to misdiagnosis and delay appropriate treatment.

The helminths described in the current article are all zoonoses, which (with the exception of *Trichobilharzia*) require human exposure to undercooked game, or to materials contaminated by animal faeces. Thus, not only prevalence in the sylvatic environment but also human lifestyle are crucial for transmission. For example, in stark contrast to the increase in trichinellosis and echinococcosis with northerly latitude in Canada (Gilbert et al., 2010; Khalid et al., 2024), anti-*Toxocara* IgG seroprevalence showed the opposite trend in that country (Bradbury and Panicker, 2020) and in Russian Federation (Akhmadishina et al., 2020), likely due to unfavourable environmental conditions for persistence of the mature infective embryonated egg and contact with humans.

Control programmes for *E. granulosus* (CE) have been successful in Iceland leading to eradication by 1979 (Saarma et al., 2023) and in human food-chain reindeer in Finland (Hirvelä-Koski et al., 2003; Oksanen and Lavikainen, 2015). For *E. multilocularis* (AE), surveillance in Nordic countries has been in place since its identification in a fox in Denmark in 2000 (Wahlström et al., 2015). In this control context, the Nunavik Trichinellosis Prevention Program, implemented in the 1997 outbreak in Nunavik in northern Quebec (Proulx et al., 2002), succeeded in preventing any further local walrus-acquired outbreaks for more than decade, despite the ongoing identification of *Trichinella*-positive walrus meat (Larrat et al., 2012).

Climate change, i.e. increase in global temperatures, has a potential impact on helminth infections in this region. Higher ambient temperatures may favour *O. felineus* transmission in Siberia by increasing cercariae survival and the amount of (non-permafrost) habitat available to *Bithynia* snails (Sripa et al., 2025). The latter consideration is also applicable to soil-dwelling embryonated eggs of *Toxocara*. Anisakid range may also be extended in polar areas (Rokicki, 2009), a zoonotic risk emphasized by the detection of these nematodes in Inuit fish and mammal food sources in the Canadian Far North (Pufall et al., 2012). We also note here the reports of cercarial dermatitis from lakes in northern Norway that experienced warm summers (Soleng and Mehl, 2011).

Herein we have brought together information from published seroprevalence studies, outbreaks, imported cases and compilations of official sources of national data to highlight this extant challenge to human health in the global Arctic and sub-Arctic. As demonstrated by these 21st-century articles, helminth infections persist across this region, potentially posing a growing threat due to warming temperatures, so should not be overlooked in assessing the global impact on human disease burden. Future directions include the need for sustained research and public recognition of the ongoing risk, aided by the refinement of serological tests to improve specificity (subgenus level) in epidemiological studies, and the broader implementation of control programmes with crucial community engagement, taking a One Health approach.

## Data Availability

No novel datasets were generated or analysed for this article. All cited references were available online via public-access resources.

## References

[ref1] Akhmadishina LV, Ruzina MN, Lukasheva MA, Kyuregyan KK, Mikhailov MI and Lukashev AN (2020) Seroprevalence and incidence of human toxocarosis in Russia. *Advances in Parasitology* 109, 419–432. 32381210 10.1016/bs.apar.2020.01.015

[ref2] Auer H, Leskowschek H, Engler J, Leitner G, Wentzel C, Wolkerstorfer W and Schneider R (2007) Epidemiology and nosology of anisakiosis, a rather rare helminthozoonosis in Central Europe-two case reports. *Wiener Klinische Wochenschrift* 119, 106–109. 17987368 10.1007/s00508-007-0866-4

[ref3] Benesh DP, Parker G and Chubb JC (2021) Life-cycle complexity in helminths: What are the benefits? *Evolution* 75, 1936–1952. 34184269 10.1111/evo.14299

[ref4] Bradbury RS and Panicker IS (2020) *Toxocara* seroprevalence in Canada - Climate, environment and culture. *Advances in Parasitology* 109, 291–316. 32381203 10.1016/bs.apar.2020.03.003

[ref5] Castrodale LJ, Beller M, Wilson JF, Schantz PM, McManus DP, Zhang LH, Fallico FG and Sacco FD (2002) Two atypical cases of cystic echinococcosis (*Echinococcus granulosus*) in Alaska, 1999. *American Journal of Tropical Medicine & Hygiene* 66, 325–327. 12139230 10.4269/ajtmh.2002.66.325

[ref6] Davidson R, Lavikainen A, Konyaev S, Schurer J, Miller A, Oksanen A, Skírnisson K and Jenkins E (2016) *Echinococcus* across the north: Current knowledge, future challenges. *Food and Waterborne Parasitology* 4, 15.

[ref7] Ducrocq J, Proulx JF, Simard M, Levesque B, Iqaluk M, Elijassiapik L, Ningiuk E, Perkins P, Jacques S and Lemire M (2020) The unique contribution of a local response group in the field investigation and management of a trichinellosis outbreak in Nunavik (Quebec, Canada). *Canadian Journal Of Public Health* 111, 31–39. 31637676 10.17269/s41997-019-00255-8 PMC7046848

[ref8] Dupouy-Camet J, Yera H, Dahane N, Bouthry E and Kapel CMO (2016) A cluster of three cases of trichinellosis linked to bear meat consumption in the Arctic. *Journal of Travel Medicine* 23. doi:10.1093/jtm/taw037 27296583

[ref9] Eskesen A, Strand EA, Andersen SN, Rosseland A, Hellum KB and Øa S (2001) Anisakiasis presenting as an obstructive duodenal tumor. A Scandinavian Case. *Scandinavian Journal of Infectious Diseases* 33, 75–76. 11234986 10.1080/003655401750064149

[ref10] Fedorova OS, Kovshirina YV, Kovshirina AE, Fedotova MM, Deev IA, Petrovskiy FI, Filimonov AV, Dmitrieva AI, Kudyakov LA, Saltykova OP, IV and Ogorodova LM (2017) *Opisthorchis felineus* infection and cholangiocarcinoma in the Russian Federation: A review of medical statistics. *Parasitology International* 66, 365–371. 27474689 10.1016/j.parint.2016.07.010

[ref11] Gilbert NL, Dare OK, Libman MD, Muchaal PK and Ogden NH (2010) Hospitalization for trichinellosis and echinococcosis in Canada, 2001-2005: The tip of the iceberg? *Canadian Journal Of Public Health* 101, 337–340. 21033550 10.1007/BF03405298 PMC6974090

[ref12] Goyette S, Cao Z, Libman M, Ndao M and Ward BJ (2014) Seroprevalence of parasitic zoonoses and their relationship with social factors among the Canadian Inuit in Arctic regions. *Diagnostic Microbiology and Infectious Disease* 78, 404–410. 24461773 10.1016/j.diagmicrobio.2013.08.026

[ref13] Hirvelä-Koski V, Haukisalmi V, Kilpelä S, Nylund M and Koski P (2003) *Echinococcus granulosus* in Finland. *Veterinary Parasitology* 111, 175–192. 12531293 10.1016/s0304-4017(02)00381-3

[ref14] Hope W, Smith-Chakmakova F and Snyder J (2020) Case of anisakiasis presenting as an Amyand hernia. *BMJ Case Reports* 13, e234822. 10.1136/bcr-2020-234822 PMC7348653 32641316

[ref15] Hotez PJ (2010) Neglected infections of poverty among the indigenous peoples of the Arctic. *PLoS Neglected Tropical Diseases* 4, e606. 20126272 10.1371/journal.pntd.0000606 PMC2811175

[ref16] Houzé S, Ancelle T, Matra R, Boceno C, Carlier Y, Gajadhar AA, and Dupouy-Camet J (2009) Trichinellosis acquired in Nunavut, Canada in September 2009: Meat from grizzly bear suspected. *Eurosurveillance* 14(44), 19383. 19941776

[ref17] Jenkins EJ, Castrodale LJ, De Rosemond SJ, Dixon BR, Elmore SA, Gesy KM, Hoberg EP, Polley L, Schurer JM, Simard M and Thompson RC (2013) Tradition and transition: Parasitic zoonoses of people and animals in Alaska, northern Canada, and Greenland. *Advances in Parasitology* 82, 33–204. 23548085 10.1016/B978-0-12-407706-5.00002-2

[ref18] Jõgi NO, Svanes C, Siiak SP, Logan E, Holloway JW, Igland J, Johannessen A, Levin M, Real FG, Schlunssen V, Horsnell WGC and Bertelsen RJ (2018) Zoonotic helminth exposure and risk of allergic diseases: A study of two generations in Norway. *Clinical and Experimental Allergy* 48, 66–77. 29117468 10.1111/cea.13055

[ref19] Khalid A, Muchaal PK and Julien DA (2024) Human echinococcosis incidence in Canada: A retrospective descriptive study using administrative hospital and ambulatory visit data, 2000-2020. *Canada Communicable Disease Report* 50, 305–311. 39267615 10.14745/ccdr.v50i09a03 PMC11392522

[ref20] Kralova-Hromadova I, Radacovska A, Cisovska Bazsalovicsova E and Kuchta R (2021) Ups and downs of infections with the broad fish tapeworm *Dibothriocephalus latus* in Europe from 1900 to 2020: Part I. *Advances in Parasitology* 114, 75–166. 34696845 10.1016/bs.apar.2021.08.008

[ref21] Kuchta R, Radacovska A, Cisovska Bazsalovicsova E and Kralova-Hromadova I (2023) Ups and downs of infections with the broad fish tapeworm *Dibothriocephalus latus* in Europe (Part II) and Asia from 1900 to 2020. *Advances in Parasitology* 122, 1–69. 37657853 10.1016/bs.apar.2023.05.001

[ref22] Larrat S, Simard M, Lair S, Belanger D and Proulx JF (2012) From science to action and from action to science: The Nunavik Trichinellosis Prevention Program. *International Journal of Circumpolar Health* 71, 18595. 22789519 10.3402/ijch.v71i0.18595 PMC3417525

[ref23] Magnaval JF, Leparc-Goffart I, Gibert M, Gurieva A, Outreville J, Dyachkovskaya P, Fabre R, Fedorova S, Nikolaeva D, Dubois D, Melnitchuk O, Daviaud-Fabre P, Marty M, Alekseev A and Crubezy E (2016) A serological survey about zoonoses in the verkhoyansk area, northeastern siberia (Sakha Republic, Russian Federation). *Vector-Borne and Zoonotic Diseases* 16, 103–109. 26807914 10.1089/vbz.2015.1828

[ref24] Magnaval JF, Tolou H, Gibert M, Innokentiev V, Laborde M, Melnichuk O, Grandadam M, Crubezy E and Alekseev A (2011) Seroepidemiology of nine zoonoses in Viljujsk, Republic of Sakha (Northeastern Siberia, Russian Federation). *Vector-Borne and Zoonotic Diseases* 11, 157–160. 20575641 10.1089/vbz.2009.0105

[ref25] Messier V, Levesque B, Proulx JF, Rochette L, Serhir B, Couillard M, Ward BJ, Libman MD, Dewailly E and Dery S (2012) Seroprevalence of seven zoonotic infections in Nunavik, Quebec (Canada). *Zoonoses Public Health* 59, 107–117. 21824376 10.1111/j.1863-2378.2011.01424.x

[ref26] Miernyk KM, Bruden D, Parkinson AJ, Hurlburt D, Klejka J, Berner J, Stoddard RA, Handali S, Wilkins PP, Kersh GJ, Fitzpatrick K, Drebot MA, Priest JW, Pappert R, Petersen JM, Teshale E, Hennessy TW and Bruce MG (2019) Human Seroprevalence to 11 Zoonotic Pathogens in the U.S. Arctic, Alaska. *Vector-Borne and Zoonotic Diseases* 19, 563–575. 30789314 10.1089/vbz.2018.2390 PMC10874833

[ref27] Møller LN (2007) Epidemiology of *Trichinella* in Greenland—occurrence in animals and man. *International Journal of Circumpolar Health* 66, 77–79. 17451137 10.3402/ijch.v66i1.18230

[ref28] Møller LN, Koch A, Petersen E, Hjuler T, Kapel CM, Andersen A and Melbye M (2010) *Trichinella* infection in a hunting community in East Greenland. *Epidemiology & Infection* 138, 1252–1256. 20144253 10.1017/S0950268810000282

[ref29] Møller LN, Krause TG, Koch A, Melbye M, Kapel CM and Petersen E (2007) Human antibody recognition of Anisakidae and *Trichinella* spp. in Greenland. *Clinical Microbiology and Infection* 13, 702–708. 17484764 10.1111/j.1469-0691.2007.01730.x

[ref30] Møller LN, Petersen E, Kapel CM, Melbye M and Koch A (2005) Outbreak of trichinellosis associated with consumption of game meat in West Greenland. *Veterinary Parasitology* 132, 131–136. 16023294 10.1016/j.vetpar.2005.05.041

[ref31] Nakhodkin SS, Pshennikova VG, Dyachkovskaya PS, Barashkov NA, Nikanorova AA, Teryutin FM, Melnichuk OA, Crubezy E, Fedorova SA and Magnaval JF (2019) A serological survey of echinococcosis, toxocariasis and trichinellosis among rural inhabitants of Central Yakutia. *International Journal of Circumpolar Health* 78, 1603550. 31046654 10.1080/22423982.2019.1603550 PMC6507814

[ref32] Oksanen A, Karssin A, Berg R, Koch A, Jokelainen P, Sharma R, Jenkins E and Loginova O (2022) Epidemiology of *Trichinella* in the Arctic and subarctic: A review. *Food and Waterborne Parasitology* 28, e00167. 35812081 10.1016/j.fawpar.2022.e00167 PMC9263860

[ref33] Oksanen A and Lavikainen A (2015) *Echinococcus canadensis* transmission in the North. *Veterinary Parasitology* 213, 182–186. 26264249 10.1016/j.vetpar.2015.07.033

[ref34] Ozeretskovskaya NN, Mikhailova LG, Sabgaida TP and Dovgalev AS (2005) New trends and clinical patterns of human trichinellosis in Russia at the beginning of the XXI century. *Veterinary Parasitology* 132, 167–171. 16081220 10.1016/j.vetpar.2005.05.056

[ref35] Pozio E (2016) Adaptation of *Trichinella* spp. for survival in cold climates. *Food and Waterborne Parasitology* 4, 9. 10.1016/j.fawpar.2022.e00154 PMC9052037 35498552

[ref36] Proulx JF, Maclean JD, Gyorkos TW, Leclair D, Richter AK, Serhir B, Forbes L and Gajadhar AA (2002) Novel prevention program for trichinellosis in inuit communities. *Clinical Infectious Diseases* 34, 1508–1514. 12015698 10.1086/340342

[ref37] Pufall EL, Jones-Bitton A, McEwen SA, Brown TM, Edge VL, Rokicki J, Karpiej K, Peregrine AS and Simard M (2012) Prevalence of zoonotic anisakid nematodes in Inuit-harvested fish and mammals from the eastern Canadian Arctic. *Foodborne Pathogens and Disease* 9, 1002–1009. 22957974 10.1089/fpd.2012.1186

[ref38] Rokicki J (2009) Effects of climatic changes on anisakid nematodes in polar regions. *Polar Science* 3, 197–201.

[ref39] Saarma U, Skirnisson K, Bjornsdottir TS, Laurimae T and Kinkar L (2023) Cystic echinococcosis in Iceland: A brief history and genetic analysis of a 46-year-old *Echinococcus* isolate collected prior to the eradication of this zoonotic disease. *Parasitology* 150, 638–643. 37161714 10.1017/S0031182023000355 PMC10261677

[ref40] Serhir B, Maclean JD, Healey S, Segal B and Forbes L (2001) Outbreak of trichinellosis associated with Arctic walruses in northern Canada, 1999. *Canada Communicable Disease Report* 27, 31–36. 11236393

[ref41] Skírnisson K (2006) *Pseudoterranova decipiens* (Nematoda, Anisakidae) larvae reported from humans in Iceland after consumption of insufficiently cooked fish. *Laeknabladid* 92, 21–25. 16400194

[ref42] Skírnisson K (2022) Human *Pseudoterranova* and *Anisakis* cases in Iceland 2004-2020. *Laeknabladid* 108, 79–83. 35103620 10.17992/lbl.2022.01.676

[ref43] Skírnisson K, Aldhoun JA and Kolárová L (2009) A review on swimmer’s itch and the occurrence of bird schistosomes in Iceland. *Journal of Helminthology* 83, 165–171. 19368747 10.1017/S0022149X09336408

[ref44] Skírnisson K and Kolarova L (2005) Swimmer’s itch in Landmannalaugar, Iceland. *Laeknabladid* 91, 729–736. 16219972

[ref45] Soleng A and Mehl R (2011) Geographical distribution of cercarial dermatitis in Norway. *Journal of Helminthology* 85, 345–352. 21070686 10.1017/S0022149X10000672

[ref46] Springer YP, Casillas S, Helfrich K, Mocan D, Smith M, Arriaga G, Mixson L, Castrodale L and McLaughlin J (2017) Two Outbreaks of Trichinellosis Linked to Consumption of Walrus Meat - Alaska, 2016-2017. *MMWR Morbidity and Mortality Weekly Report* 66, 692–696. 28683055 10.15585/mmwr.mm6626a3 PMC5726240

[ref47] Sripa B, Yurlova N, Suwannatrai AT, Serbina E, Tangkawattana S, Sayasone S and Varnakovida P (2025) Potential impact of climate change on *Opisthorchis viverrini* and *Opisthorchis felineus* transmission in Eurasia. *Acta Tropica* 263, 107574. 40037476 10.1016/j.actatropica.2025.107574

[ref48] Stadlbauer V, Haberl R, Langner C, Krejs GJ and Eherer A (2005) Annoying vacation souvenir: Fish tapeworm (*Diphyllobothrium* sp.) infestation in an Austrian fisherman. *Wiener Klinische Wochenschrift* 117, 776–779. 16416360 10.1007/s00508-005-0463-3

[ref49] Usmanova NM and Kazakov VI (2010) The DL1 repeats in the genome of *Diphyllobothrium latum*. *Parasitology Research* 107, 449–452. 20440623 10.1007/s00436-010-1889-8

[ref50] Uspensky A, Bukina L, Odoevskaya I, Movsesyan S and Voronin M (2019) The epidemiology of trichinellosis in the Arctic territories of a Far Eastern District of the Russian Federation. *Journal of Helminthology* 93, 42–49. 29382411 10.1017/S0022149X18000020

[ref51] Wahlström H, Enemark HL, Davidson RK and Oksanen A (2015) Present status, actions taken and future considerations due to the findings of *E. multilocularis* in two Scandinavian countries. *Veterinary Parasitology* 213, 172–181. 26362495 10.1016/j.vetpar.2015.07.037

[ref52] Wicht B, Ruggeri-Bernardi N, Yanagida T, Nakao M, Peduzzi R and Ito A (2010) Inter- and intra-specific characterization of tapeworms of the genus *Diphyllobothrium* (Cestoda: Diphyllobothriidea) from Switzerland, using nuclear and mitochondrial DNA targets. *Parasitology International* 59, 35–39. 19800982 10.1016/j.parint.2009.09.002

[ref53] Yossepowitch O, Gotesman T, Assous M, Marva E, Zimlichman R and Dan M (2004) Opisthorchiasis from imported raw fish. *Emerging Infectious Diseases* 10, 2122–2126. 15663848 10.3201/eid1012.040410 PMC3323388

